# Supplementary material for: 8-Oxoguanine Disrupts G-Quadruplex DNA Stability and Modulates FANCJ AKKQ Peptide Binding
Source: Molecules. 2025 Aug 20;30(16):3424. doi: 10.3390/molecules30163424 (PMC12388576; doi:10.3390/molecules30163424)
Supplement: Supplementary file 1 [file molecules-30-03424-s001.zip › molecules-3782702-supplementary.pdf]

8-Oxoguanine Disrupts G-Quadruplex DNA Stability and Modulates FANCJ AKKQ Peptide Binding

Supplementary Material

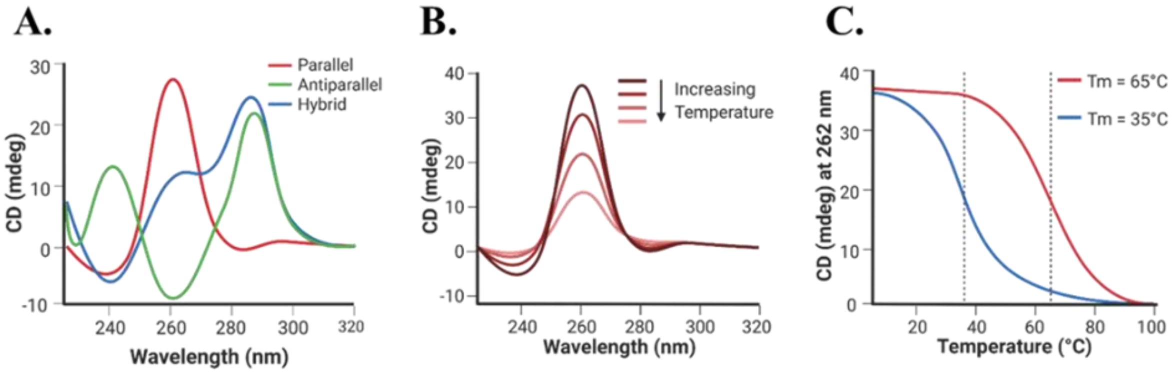

Figure S1. CD spectroscopy cartoons. (A) Graphic representation of CD spectra for parallel, antiparallel, and hybrid G4 conformations. Parallel conformations have a maximum at 262 nm and a minimum at 240 nm. Antiparallel conformations give two peak signals near 295 nm and 240 nm, and a minimum at 260 nm. Hybrid conformations show a peak at 295 nm, a shoulder around 260 nm, and a minimum at 240 nm. (B) Graphic representation of the CD spectra of a parallel G4 conformation as a function of temperature. (C) Graphic representation of a thermal melt graph of two G4s.

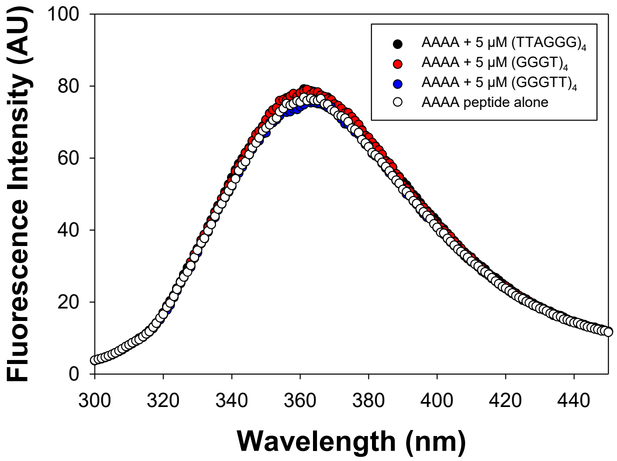

Figure S2. Fluorescence titrations performed with a FANCJ AAAA peptide. A peptide was synthesized with the AKKQ motif modified to AAAA (129-PEKTTLAAKLSAAAAASIW-147) to serve as a negative control. Fluorescence spectroscopy experiments were repeated with the peptide alone (O), or in the presence of 5 μM of $(TTAGGG)_4$ (●), $(GGGT)_4$ (●), or $(GGGTT)_4$ (●).

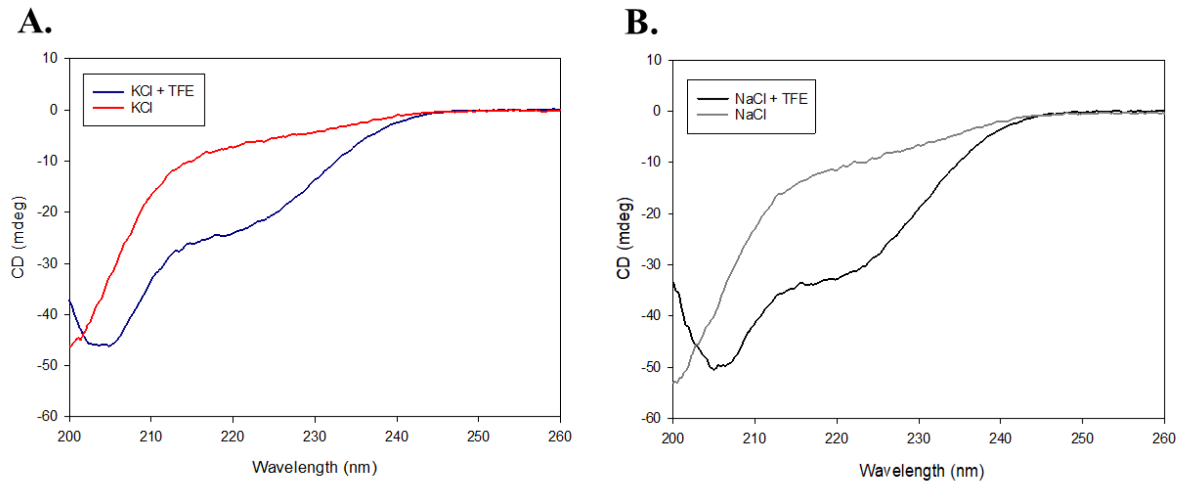

Figure S3. Experiments performed in the presence and absence of 20% (v/v) trifluoroethanol. Circular dichroism (CD) data was collected on a JASCO J-815 spectropolarimeter (JASCO Inc.; Easton, MD, USA) equipped with a PTC-423S peltier system. Protein samples were analyzed either in trifluoroethanol-containing buffer (20 mM boric acid pH 7.5, 150 mM KCl or NaCl, 5% (v/v) glycerol, and 20% (v/v) trifluoroethanol) or buffer without trifluoroethanol (20 mM boric acid pH 7.5, 150 mM KCl or NaCl, and 5% (v/v) glycerol). The presence of trifluoroethanol was used to evaluate differences in protein secondary structure based on the corresponding salt condition. Spectra of FANCJ AKKQ were collected from 260 nm to 200 nm at 25 °C. Five traces were collected for each sample, and reference scans of buffer without protein were subtracted from the averaged CD signal. (A) CD spectra of FANCJ AKKQ taken from 260 to 200 nm in the presence (blue) and absence (red) of trifluoroethanol in 150 mM KCl. (B) CD Spectra of FANCJ AKKQ taken from 260 to 200 nm in the presence (black) and absence (gray) of trifluoroethanol in 150 mM NaCl. Upon the addition of trifluoroethanol in either salt condition, a helical-like conformation appears.
